# Association between excessive screen time and school-level proportion of no family rules among elementary school children in Japan: a multilevel analysis

**DOI:** 10.1265/ehpm.23-00268

**Published:** 2024-03-16

**Authors:** Masaaki Yamada, Michikazu Sekine, Takashi Tatsuse

**Affiliations:** Department of Epidemiology and Health Policy, School of Medicine, University of Toyama, Toyama, Japan

**Keywords:** Addiction, Algorithms, Environment, Family rules, Problematic, Gaming disorder

## Abstract

**Background:**

Excessive screen time (ST) in children is a global concern. We assessed the association between individual- and school-level factors and excessive ST in Japanese children using a multilevel analysis.

**Methods:**

A school-based cross-sectional study was conducted in Toyama, Japan in 2018. From 110 elementary schools in Toyama Prefecture, 13,413 children in the 4th-6th grades (boys, 50.9%; mean, 10.5 years old) participated. We assessed lifestyle, recreational ST (not for study use), psychological status, and school and family environment including family rules. We defined ≥3 hours ST as excessive. We calculated the school-level proportions of no family rules and divided them into four categories (<20%, 20% to <30%, 30% to <40%, and ≥40%). A modified multilevel Poisson regression analysis was performed.

**Results:**

In total, 12,611 children were included in the analysis (94.0%). The average school-level proportion of those with no family rules was 32.1% (SD = 9.6). The prevalence of excessive ST was 29.9% (34.9% in boys; 24.8% in girls). The regression analysis showed that excessive ST was significantly associated with both individual-level factors, such as boys (adjusted prevalence ratio (aPR); 1.39), older grades (aPR; 1.18 for 5th grades and 1.28 for 6th grades), late wakeup (aPR; 1.13), physical inactivity (aPR; 1.18 for not so much and 1.31 for rarely), late bedtime (aPR; 1.43 for 10 to 11 p.m. and 1.76 for ≥11 p.m.), frequent irritability (aPR; 1.24 for sometimes and 1.46 for often), feelings of school avoidance (aPR; 1.17 for sometimes and 1.22 for often), infrequent child-parental interaction (aPR; 1.16 for rare and 1.21 for none), no family rules (aPR; 1.56), smartphone ownership (aPR; 1.18), and the school-level proportion of no family rules (aPR; 1.20 for 20% to <30%, 1.29 for 30% to <40%, and 1.43 for ≥40%, setting <20% as reference).

**Conclusion:**

Besides individual factors, a higher school-level proportion of no family rules seemed influential on excessive ST. Increasing the number of households with family rules and addressing individual factors, could be deterrents against excessive ST in children.

## Graphical Abstract

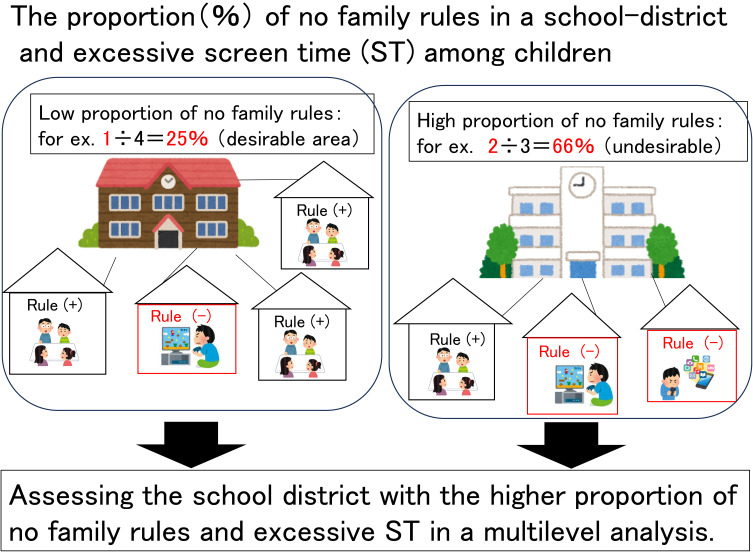

## Introduction

Excessive screen time (ST) for recreational use by children is a global concern, and has been associated with a wide range of poor health outcomes, including obesity [1], depression [2], developmental delays [3], low academic achievement [4], and Internet addiction [5]. In 2004, the Japanese Pediatric Association proposed that ST in all growing children should be reduced to nurture whole-some family relationships [6]. However, the coronavirus disease 2019 (COVID-19) has dramatically changed children’s lives and their relationship with screen devices. Schools were closed and people’s social activities were restricted for disease control in Japan, in 2020. Therefore, digitalization in education system was accelerated under the policy of “GIGA” school project [7], which stands for Global and Innovation Gateway for All. Digitalization offers many educational and social benefits; however, the current situation has caused increased concern for parents and health providers regarding excessive ST for recreational use in children.

ST in children has previously been associated with their lifestyles, such as sleep deficiency and physical inactivity [8, 9], and family environments [10, 11], including parental rules to limit ST, parental internet time, co-viewing with parents, and the presence of screen devices in their bed room [8]. Among them, parental rules appear to be one of the key factors that influence the amount of ST in children. One task force for reducing ST stated that family based support was the most common and important component in changing screen behaviors among children aged 13 years and younger [12]. Preventive education on excessive ST is crucial in the early childhood stage [13]. We previously reported that children who did not have rules to restrict ST at home were more likely to be associated with prolonged ST and pathological Internet use and gaming [5, 10, 14]. These findings were in line with other reports demonstrating the importance of family rules as an individual component [8, 11, 15]. However, no studies have explored family rules as an environmental factor.

The extremely rapid Internet connectivity has enabled children to engage in online gaming together or engage in prolonged social interaction, creating and sharing content while at home. This interconnectedness between friends might be a hurdle to restricting ST for parents because these online activities are difficult to restrict and follow family rules. Therefore, besides individual factors, environmental (school-level) factors should also be explored because they seem to strongly influence children’s ST.

Several prior studies have examined school-level factors and children’s ST [16, 17]. Abdel et al. showed that in a school district, a higher proportion of non-White students and a lower proportion of parents with college education were associated with longer individual ST [16]. From the perspective of public health, we believe that identifying modifiable school-level factors was desirable for parents and health professionals to provide a healthy school environment. We hypothesized that promoting family rules would be feasible in every school and that a higher school-level proportion of family rules would work as a preventive strategy toward excessive ST in individual children. Therefore, we aimed to explore the association between individual factors, school-level proportion of family rules, and excessive ST among elementary school children in Japan.

## Methods

### Study design and participants

This school-based cross-sectional study, the Toyama Safe Internet Use Project, was conducted in Toyama, Japan in 2018 [5, 14]. Toyama Prefecture is located in central Honshu and has a population of approximately 1 million. Responding to an invitation from Toyama Prefecture Education board, 110 out of 185 elementary schools (61.1% of elementary schools in Toyama, as of 2018) participated in this project. In total, 13,413 children in the 4th to 6th grades were included in our study. Self-reported anonymous questionnaires were distributed to all the school children via school teachers.

### Individual measures and main outcome

Our questionnaires inquired about the children’s basic characteristics, lifestyles, psychological status, school and family environments, and ST. Lifestyle variables included wake-up time, daily breakfast, physical activity, and sleep habits. Responses to the wake-up time and breakfast were dichotomized: “<7:00 a.m.” or “≥7:00 a.m.” and “every day” or “skipping breakfast,” respectively. Physical activity responses were answered at three levels: “often,” “not so much,” or “rarely to almost never.” The validity of the lifestyle questions regarding physical activity and sleep habits were examined as in previous studies, and they demonstrated good agreement in subjective and objective measures [18, 19]. In these studies, frequent physical activity was significantly correlated with daily energy expenditure, mean steps, and mean activity count on the Actiwatch (p < 0.05 for a linear trend test). The correlation between subjective and objective records was 0.97 (p < 0.001) for assumed amount of sleep. To asses psychological status, we inquired about frequency of feelings of irritability (“How often do you feel irritated?”) and the frequency of school avoidance (“How often do you feel like you are reluctant to go to school?”).

Regarding the school environment, information about close friends (“How many close friends do you have in real life?”) and subjective academic performance (“Do you understand school lectures well?”) were asked. The response of academic performance was assessed on a five-point scale and collapsed into three categories: “high,” “middle,” and “low.” Subjective academic performance has previously been reported to be generally accurate [20], and has been used widely as a feasible surrogate variable [4, 21, 22]. Regarding family environment, we asked about the setting of family rules for restricting ST, ownership of smartphone or cell-phone, and about child-parent interactions (“How often do you usually interact with your parents?”). As the main outcome, we asked about daily recreational ST on a weekday, which included TV and DVD viewing, videogame playing, and Internet browsing only for recreational use. The American Academy of Pediatrics recommends children’s ST to be within 2 hours per day, as prolonged ST causes childhood obesity and sleep shortage, while negatively affect school performance [6]. Therefore, children’s ST was divided into “<3 h” or “≥3 h”, and the latter was defined as “excessive” in this study.

### School-level proportion of no family rule

From the answers to family rules (yes or no), we calculated the proportion of those having no family rules among 110 school districts and defined each school proportion as a school-level variable. The school-level proportion was divided into 4 levels: <20%, 20% to <30%, 30% to <40%, and ≥40%.

### Statistical analysis

Descriptive analyses were performed for all variables and ST according to sex. We employed a modified two-level (individual child and school district) Poisson regression analysis with a random intercepts model to explore the prevalence ratios (PR) of excessive ST [23]. Univariable (crude) PR and multivariable aPR in individual-level and a fully adjusted two-level model, as well as 95% confidence intervals (95% CI) were calculated. In the multivariable model, all individual variables including basic characteristics, lifestyles, psychological status, as well as school and family environments, were simultaneously analyzed using a forced entry method. Moreover, the school-level variance and the median rate ratio were shown both in the null and fully adjusted models. To explore differences according to sex, additional analyses stratified by sex were conducted.

As a post hoc test, we included a cross-level interaction term between individual family rules and the school-level proportion of family rules. However, Akaike’s information criterion (AIC) of the model with the cross-level interaction term did not fit better than the model without the interaction term (15548.6 vs 15546.1); therefore, we employed the model without the cross-level interaction term in this study. Data analyses were performed using STATA software (version 16.0; STATA Corporation, College Station, TX, USA). Statistical significance was set at p < 0.05.

## Results

A total of 13,092 children returned the questionnaire (response rate: 97.6%), and 12,611 (94.0%) children who responded to all relevant questions were included in our analyses (M_age_: 10.5 years, SD: 0.96). The prevalence of excessive ST (≥3 h) was 29.9% (34.9% in boys, 24.8% in girls). Table 1 describes the distribution of the basic characteristics according to sex. More boys tended to get up late, skip breakfast, be physically active, have frequent feelings of school avoidance, have family rules, and have longer ST than girls. The girls interacted more frequently with their parents.

**Table 1:** Characteristics of children (n = 12,611)

		**Total**	**Boys**	**Girls**	**p**
		**%**	**n = 6399** **(%)**	**n = 6212** **(%)**	**Chi-square test**
Grades	4	32.6	2116(33.1)	2001(32.2)	0.500
	5	33.4	2111(33.0)	2102(33.8)	
	6	33.9	2172(33.9)	2109(34.0)	
Wakeup time on weekdays	≥7:00 a.m.	4.4	341(5.3)	217(3.5)	<0.001
Breakfast	skipping / every day	8.8	594(9.3)	512(8.2)	0.039
Physical activity	often	73.0	5055(79.0)	4155(66.9)	<0.001
	not so much	23.0	1123(17.5)	1773(28.5)	
	rarely	4.0	221(3.5)	284(4.6)	
Bedtime on weekdays	<10 p.m.	66.1	4278(66.9)	4052(65.2)	0.059
	10 to 11 p.m.	26.9	1664(26.0)	1732(27.9)	
	≥11 p.m.	7.0	457(7.1)	428(6.9)	
Irritability	rare	34.0	2256(35.3)	2034(32.7)	0.012
	sometimes	44.2	2771(43.3)	2803(45.1)	
	often	21.8	1372(21.4)	1375(22.1)	
Feelings of school avoidance	rare	59.6	3640(56.9)	3870(62.3)	<0.001
	sometimes	29.7	1922(30.0)	1828(29.4)	
	often	10.7	837(13.1)	514(8.3)	
Close friends in real life	many	64.3	4125(64.5)	3987(64.2)	0.185
	some	29.6	1863(29.1)	1868(30.1)	
	scarce or none	6.1	411(6.4)	357(5.7)	
Academic performance in school	high	86.7	5537(86.5)	5398(86.9)	0.285
	middle	9.1	573(9.0)	570(9.2)	
	low	4.2	289(4.5)	246(4.0)	
Interaction with parents	often or sometime	76.3	4368(68.3)	5249(84.5)	<0.001
	rare	17.1	1393(21.8)	761(12.3)	
	none	6.7	638(10.0)	202(3.3)	
Family rules for screen time (individual)	no	31.5	2089(32.6)	1880(30.3)	0.004
Smartphone ownership	yes	20.5	1240(19.4)	1341(21.6)	<0.001
Screen time (not study use) on weekdays	<2 h	45.8	2595(40.6)	3178(51.2)	<0.001
	2 h to <3 h	24.3	1573(24.6)	1493(24.0)	
	3 h to <4 h	14.8	1053(16.5)	816(13.1)	
	≥4 h	15.1	1178(18.4)	725(11.7)	

Table 2 shows the school-level proportion of those with no family rules among the 110 elementary schools. The mean proportion was 32.1%, ranging from 10.0% to 64.2% with an SD of 9.6.

**Table 2:** School-level proportion of no family rules (n = 110 schools)

**Mean and range of school-level proportion**			

**Mean**	**Standard deviation**	**Minimum**	**Maximum**
32.1%	9.6	10.0%	64.2%



**Proportion of ** **no rules**	**Number of ** **schools**	**%**	**Number of children** **(individual-level)**

<20%	10	9.1	956
20% to <30%	34	30.9	4414
30% to <40%	50	45.5	6103
≥40%	16	15.5	1138

Total	110	100	12611

The results of the two-level Poisson regression analysis exploring the association between individual variables, school-level proportion of no family rules, and excessive ST are shown in Table 3. In the two-level full adjusted model, boys (aPR 1.39; 95% CI 1.30–1.48), older grade (5th grade: aPR 1.18; 95% CI 1.08–1.30 and 6th grade: aPR 1.28; 95% CI 1.16–1.42), late wakeup (≥7 a.m.: aPR 1.13; 95% CI 1.04–1.23), physical activity (not so much: aPR 1.18; 95% CI 1.11–1.25 and rarely: aPR 1.31; 95% CI 1.17–1.47), late bedtime (10 to 11 p.m.: aPR 1.43; 95% CI 1.35–1.52 and ≥11 p.m.: aPR 1.76; 95% CI 1.62–1.91), feeling of irritability (sometimes: aPR 1.24; 95% CI 1.16–1.32 and often: aPR 1.46; 95% CI 1.34–1.58), feelings of school avoidance (sometimes: aPR 1.17; 95% CI 1.13–1.25 and often: aPR 1.22; 95% CI 1.10–1.32), close friends in real life (scarce or none: aPR 0.81; 95% CI 0.73–0.91), interaction with parents (rare: aPR 1.16; 95% CI 1.09–1.24 and none: aPR 1.21; 95% CI 1.13–1.30), no family rules at home (aPR 1.56; 95% CI 1.47–1.64), and smartphone ownership (aPR 1.18; 95% CI 1.11–1.25) were significantly associated with excessive ST. Besides individual factors, the school-level proportion of no family rules showed significant associations (“<30%”: aPR 1.20; 95% CI 1.02–1.41, “<40%”: aPR 1.29; 95% CI 1.09–1.51 and “≥40%”: aPR 1.43; 95% CI 1.19–1.74). The two-level Poisson regression analysis confirmed that the PR of excessive ST only slightly varied between school-levels because the median rate ratio was small (1.25) in both the null model and the fully adjusted model (1.14).

**Table 3:** Modified Poisson regression analyses on excessive screen time (n = 12,611)

		**Excessive ST**	**Individual-level univariable (crude)**	**Individual-level multivariable**	**Null-model**	**Two-level multivariable (full adjusted)**	
		**%**	**PR (95% CI)**	**aPR (95% CI)**		**aPR (95% CI)**	**p**
					**Fixed effect**		

Sex	boys/ girls	34.9/24.8	**1.40 (1.31–1.49)**	**1.38 (1.31–1.46)**		**1.39 (1.30–1.48)**	**<0.001**
Grades	4	23.4	1	**1**		1	
	5	30.5	**1.29 (1.19–1.40)**	**1.18 (1.10–1.26)**		**1.18 (1.08–1.30)**	**<0.001**
	6	35.6	**1.51 (1.40–1.63)**	**1.28 (1.20–1.37)**		**1.28 (1.16–1.42)**	**<0.001**
Wakeup time on weekdays	≥7:00/<7:00 a.m.	49.5/29.0	**1.70 (1.50–1.91)**	**1.13 (1.03–1.23)**		**1.13 (1.04–1.23)**	**0.004**
Breakfast,	skipping / every day	43.1/28.6	**1.51 (1.37–1.66)**	1.00 (0.93–1.08)		1.01 (0.93–1.09)	0.827
Physical activity	often	27.7	**1**	**1**		**1**	
	not so much	34.3	**1.24 (1.15–1.33)**	**1.17 (1.11–1.25)**		**1.18 (1.11–1.25)**	**<0.001**
	rarely	45.3	**1.61 (1.41–1.84)**	**1.31 (1.18–1.45)**		**1.31 (1.17–1.47)**	**<0.001**
Bedtime on weekdays	<10 p.m.	22.9	**1**	1		**1**	
	10 to 11 p.m.	39.2	**1.71 (1.59–1.83)**	**1.45 (1.37–1.53)**		**1.43 (1.35–1.52)**	**<0.001**
	≥11 p.m.	60.7	**2.65 (2.42–2.92)**	**1.77 (1.64–1.90)**		**1.76 (1.62–1.91)**	**<0.001**
Irritability	rare	21.5	**1**	**1**		**1**	
	sometimes	30.5	**1.41 (1.30–1.53)**	**1.24 (1.16–1.33)**		**1.24 (1.16–1.32)**	**<0.001**
	often	42.2	**1.95 (1.79–2.13)**	**1.46 (1.36–1.58)**		**1.46 (1.34–1.58)**	**<0.001**
Feelings of school avoidance	rare	24.0	1	**1**		1	
	sometimes	35.7	**1.48 (1.39–1.60**)	**1.18 (1.11–1.25)**		**1.17 (1.13–1.25**)	**<0.001**
	often	46.8	**1.97 (1.81–2.16)**	**1.23 (1.14–1.33)**		**1.22 (1.10–1.32)**	**<0.001**
Close friends	many	28.5	1	1		**1**	
	some	32.5	**1.13 (1.06–1.22)**	0.94 (0.89–1.00)		0.94 (0.87–1.01)	0.064
	scarce or none	31.9	1.11 (0.98–1.27)	**0.81 (0.73–0.91)**		**0.81 (0.73–0.91)**	**0.001**
Academic performance in school	high	28.0	1	1		1	1
	middle	40.7	**1.45 (1.33–1.61)**	1.05 (0.97–1.13)		1.05 (0.96–1.13)	0.277
	low	45.7	**1.60 (1.41–1.83)**	1.08 (0.99–1.19)		1.08 (0.98–1.19)	0.146
Interaction with parents	often or sometime	26.3	1	1		1	
	rare	38.6	**1.45 (1.35–1.58)**	**1.17 (1.10–1.25)**		**1.16 (1.09–1.24)**	**<0.001**
	none	48.7	**1.84 (1.66–2.04)**	**1.23 (1.13–1.33)**		**1.21 (1.13–1.30)**	**0.001**
Family rules for screen time	no/yes	45.2/22.9	**1.98 (1.86–2.11)**	**1.59 (1.51–1.67)**		**1.56 (1.47–1.64)**	**<0.001**
Smartphone ownership	yes/no	38.9/27.6	**1.41 (1.32–1.52)**	**1.17 (1.11–1.24)**		**1.18 (1.11–1.25)**	**<0.001**
School-level proportion of no family rules	<20% (ref)	19.8				1	
	20% to <30%	27.1				**1.20 (1.02–1.41)**	**0.027**
	30% to <40%	32.2				**1.29 (1.09–1.51)**	**0.011**
	≥40%	37.2				**1.43 (1.19–1.74)**	**0.001**
					**Random effect**		
School-level variance (SE)					0.053 (0.013)	0.018 (0.007)	
Median rate ratio					1.25	1.14	

ST, screen time; PR, prevalence ratio; aPR, adjusted prevalence ratio; CI, confidence interval; SE, standard error.

The two-level Poisson regression analyses were stratified by sex (Table 4). Results showed similar trends between sexes in almost all variables; however, the school-level proportion of no family rules was more strongly associated with excessive ST in girls than in boys.

**Table 4:** Modified Poisson regression analyses on excessive screen time by sex

		**Boys, n = 6399**			**Girls, n = 6212**		

		**Excessive ** **ST**	**Two-level multivariable ** **(full adjusted)**		**Excessive ** **ST**	**Two-level multivariable ** **(full adjusted)**	
		**%**	**aPR (95% CI)**	**p**	**%**	**aPR (95% CI)**	**p**
**Fixed effect**							

Grades	4	29.1	**1**		17.3	**1**	
	5	35.6	**1.13 (1.03–1.25)**	**0.011**	25.3	**1.27 (1.10–1.46)**	**0.001**
	6	39.7	**1.19 (1.07–1.33)**	**0.002**	31.4	**1.44 (1.25–1.67)**	**<0.001**
Wakeup time on weekdays	≥7:00/<7:00 a.m.	52.8/33.9	1.06 (0.94–1.20)	0.317	44.2/24.1	**1.30 (1.12–1.51)**	**0.001**
Breakfast,	skipping / every day	46.1/33.7	0.97 (0.89–1.06)	0.482	39.6/23.5	1.05 (0.91–1.21)	0.476
Physical activity	often	32.5	**1**		21.8	**1**	
	not so much	42.3	**1.20 (1.12–1.28)**	**<0.001**	29.2	**1.14 (1.04–1.26)**	**0.007**
	rarely	50.2	**1.24 (1.06–1.46)**	**0.008**	41.5	**1.36 (1.18–1.56)**	**<0.001**
Bedtime on weekdays	<10 p.m.	26.9	**1**		18.6	**1**	
	10 to 11 p.m.	46.0	**1.45 (1.34–1.56)**	**<0.001**	32.6	**1.42 (1.29–1.56)**	**<0.001**
	≥11 p.m.	68.9	**1.82 (1.64–2.02)**	**<0.001**	51.9	**1.64 (1.46–1.85)**	**<0.001**
Irritability	rare	26.2	**1**		16.3	**1**	
	sometimes	36.4	**1.25 (1.15–1.36)**	**<0.001**	24.3	**1.24 (1.10–1.40)**	**0.001**
	often	46.0	**1.33 (1.22–1.44)**	**<0.001**	38.3	**1.65 (1.44–1.90)**	**<0.001**
Feelings of school avoidance	rare	28.8	1		19.5	1	
	sometimes	39.1	**1.12 (1.05–1.20)**	**0.001**	32.1	**1.23 (1.11–1.36)**	**0.002**
	often	51.5	**1.23 (1.11–1.36)**	**<0.001**	39.1	**1.19 (1.03–1.38)**	**<0.001**
Close friends	many	34.0	1		22.8	**1**	
	some	35.9	**0.91 (0.84–0.98)**	**0.016**	29.2	0.98 (0.89–1.08)	0.659
	scarce or none	38.7	**0.82 (0.72–0.94)**	**0.003**	24.1	**0.79 (0.65–0.95)**	**0.015**
Academic performance in school	high	32.8	1	1	23.1	1	1
	middle	46.9	1.05 (0.95–1.16)	0.372	34.4	1.04 (0.92–1.18)	0.530
	low	50.5	1.08 (0.97–1.21)	0.169	39.4	1.07 (0.90–1.28)	0.462
Interaction with parents	often or sometime	30.7	1		22.7	1	
	rare	40.4	**1.14 (1.06–1.22)**	**<0.001**	35.2	**1.23 (1.07–1.40)**	**0.002**
	none	51.1	**1.24 (1.14–1.35)**	**<0.001**	41.1	**1.20 (1.01–1.43)**	**0.039**
Family rules for screen time	no/yes	51.6/26.8	**1.55 (1.45–1.66)**	**<0.001**	38.2/19.0	**1.56 (1.43–1.70)**	**<0.001**
Smartphone ownership	yes/no	45.3/32.4	**1.15 (1.08–1.23)**	**<0.001**	32.9/22.6	**1.20 (1.09–1.31)**	**<0.001**
School-level proportion of no family rules	<20% (ref)	24.4	1		15.4	1	
	20% to <30%	31.1	1.10 (0.88–1.38)	0.411	22.8	**1.31 (1.08–1.58)**	**0.027**
	30% to <40%	37.5	1.20 (0.96–1.51)	0.113	26.8	**1.38 (1.14–1.67)**	**0.011**
	≥40%	43.9	**1.36 (1.07–1.73)**	**0.011**	30.2	**1.49 (1.17–1.90)**	**0.001**
**Random effect**							
School-level variance (SE)			0.008 (0.006)			0.025 (0.012)	
Median rate ratio			1.09			1.16	

ST, screen time; PR, prevalence ratio; aPR, adjusted prevalence ratio; CI, confidence interval; SE, standard error.

## Discussion

In this large-scale multilevel study among Japanese elementary school children, we found that both individual-level factors, including undesirable lifestyle, psychological status; school and family environments; and a higher school-level proportion of no family rules were associated with excessive ST. In addition, a dose-response relationship was observed between school-level family rules and excessive ST, the relationship being stronger in girls. Our results demonstrate the importance of school-level and individual household strategies to restrict excessive ST in children.

In our study, the prevalence of excessive ST was 29.9% (34.9% in boys and 24.8% in girls), which was slightly lower than that of the national report in 2018 (38.1% in boys and 28.9% in girls) [24]. Some review articles demonstrated that male sex and older adolescents were associated with Internet gaming disorder or excessive gaming [25, 26]. Similarly, boys and older children in our study were more likely to have excessive ST. Boys’ enthusiasm in gaming was considered to contribute to the sex difference.

We found that undesirable lifestyles such as late wakeup, physical inactivity, late bedtime on weekdays, were associated with excessive ST, and were consistent with previous studies [9, 27, 28]. Time consumption due to excessive ST may be taking away from exercise and sleep time. Some children might have intrinsically evening-type circadian rhythms, however, given the highest aPR for late bedtime (≥11 p.m.: aPR; 1.76 in Table 3) in our study, having healthy sleep habits should be particularly encouraged for children to decrease ST.

In our study, significant associations were observed between excessive ST and psychological status including frequent feelings of irritability and school avoidance. These results were consistent with other studies, showing an association with poor mental status such as depression, ADHD-related behaviors, and even suicidal behavior [9, 29–31]. Causal relationship between excessive ST and poor mental status or developmental disorders is controversial and difficult to clarify, even in longitudinal studies [32–34]. However, many advanced Internet technologies (IT), such as machine learning and algorithms that can captivate or engage users (children), have been introduced in the IT industry. Algorithms that are designed to drive sales by companies or the Internet industry also work to captivate uses, thus, these algorithms are called “addictive algorithms [35].” Social networking service and online game industry can keep track of what users look at and how long they look at it; the industry can then augment users’ engagement by knowing what posts (articles) or gaming content users need most at that moment [36]. The most addictive and engaging content on the Internet is known to cause anger. Such content is more likely to be put at the top of user’s post or screen [37]. Given these addictive algorithms working endlessly, children with excessive ST are exposed to obnoxious or violent content for long time, thus, resulting in their poor mental health. Thus, excessive ST can cause mental health problems in children.

Family environment, including infrequent child-parent interactions, smartphone ownership, and family rules at an individual household (individual level), were associated with excessive ST in our study. Our findings are in line with those of previous studies [24, 38–40]. Family environment, especially child-parent interactions, are important for children. Parental engagement in child rearing plays an important role in the development of children’s emotional and social development, and parental distractions by media use are known to have short- and long-term negative effects [41]. Regarding family rules, we only asked about the existence of family rules, not specifying the kind of rules. The American Academy of Pediatrics developed Family Media Use Plan, and recommends to let children have 2 hours or less of sedentary screen time daily and monitor their media contents, to promote children to get the recommended amount of daily physical activity (1 hour) and adequate sleep (8–12 hours, depending on age), and designate media-free times together (eg, family dinner) and media-free locations (eg, bedrooms) in homes [41]. Specifying family rules along with the recommendations would be useful to explore more protective or feasible rules to decrease ST of children in the future.

Beside family rules at the individual household, we found that the school-level proportion of no family rules was significantly associated with children’s excessive ST. Children who lived in the school district where many families did not have family rules (e.g., the proportion was ≥20% in Table 3) were more likely to have excessive ST. In addition, the association was stronger in girls than in boys (Table 4). This may stem from the fact that girls are more likely to be absorbed in social networking and texting [9, 25]. There have rarely been studies showing the association between school-level (environmental) factors and ST among children [16, 17]. To the best of our knowledge, this is the first study to clarify the association with the proportion of family rules as a school-level variable, after simultaneously adjusting for individual factors. We can infer that environmental atmosphere affects individual screen behaviors among children. Therefore, we recommend that preventive strategies, including setting family rules to restrict ST, be implemented in the entire school district and not only in individual households. These can be more effective to girls, given their strong association between no family rules and excessive ST.

The study’s strengths lie in it being a large-scale study with a high participation rate; however, there are several limitations that must be considered. First, it was a self-reported survey, which may be subject to recall and under-reporting biases. Second, our study participants were from only one prefecture in Japan, which limits the generalizability of our findings. Third, as our study was cross-sectional, causality could not be elucidated; a reverse causality could occur between dependent and independent variables. However, we demonstrated a dose-response relationship between the school-level proportion of no family rules and excessive ST. Moreover, it seems practical to imagine that a higher proportion of no family rules in a school district can lead to increased individual ST. The opposite is considered impractical. Although longitudinal studies are needed to clarify causality, school-based preventive strategies as well as individual strategies are desirable for decreasing children’s ST.

In conclusion, our large-scale study found that in addition to individual-level factors, including undesirable lifestyle, psychological status, and school and family environments, a higher school-level proportion of no family rules was significantly associated with excessive ST. Therefore, we suggest that the environment surrounding a school could affect individual ST. Our results demonstrate the importance of school-level as well as individual-level prevention strategies to restrict children’s excessive ST. Further studies are needed to delineate the specific family rules by which we can encourage more children to have family rules.

## Abbreviations

ADHD: Attention-Deficit / Hyperactivity Disorder
AIC: Akaike information criterion
aPR: adjusted prevalence ratio
CI: Confidence interval
COVID-19: the coronavirus disease 2019
PR: prevalence ratio
ST: screen time

## Declarations

### Data availability

The data are from the Toyama Safe Internet Use Project and the dataset has ethical restrictions because it includes information on elementary schools and children. Thus, the data are not open for public use. Several parts of dataset analyzed during the current study are available from the corresponding author on reasonable request.
